# Silver Carp (*Hypophthalmichthys molitrix*) Scales Collagen Peptides (SCPs): Preparation, Whitening Activity Screening and Characterization

**DOI:** 10.3390/foods12071552

**Published:** 2023-04-06

**Authors:** Xiao-Yan Zu, Mei-Jin Li, Guang-Quan Xiong, Jun Cai, Tao Liao, Hai-Lan Li

**Affiliations:** 1 Key Laboratory of Cold Chain Logistics Technology for Agro-Product (Ministry of Agriculture and Rural Affairs), Institute of Agro-Products Processing and Nuclear Agricultural Technology, Hubei Academy of Agricultural Sciences, Wuhan 430064, China; zuxiaoyan@hbaas.com (X.-Y.Z.); lmj602@hbut.edu.cn (M.-J.L.); xiongguangquan@163.com (G.-Q.X.); oriswa@sina.com (T.L.); 2 Key Laboratory of Fermentation Engineering (Ministry of Education), Hubei Provincial Cooperative Innovation Center of Industrial Fermentation, Hubei Key Laboratory of Industrial Microbiology, Hubei University of Technology, Wuhan 430068, China; hgdcaijun@hbut.edu.cn

**Keywords:** scale, collagen peptide, whitening, characterization, identification

## Abstract

This study involves the preparation of scale collagen peptides (SCPs) with whitening activity from silver carp (*Hypophthalmichthys molitrix*) and their characterization and peptide sequence identification. In this article, scanning electron microscopy (SEM) was used to observe structure changes of sliver carp scales; enzymatic hydrolysis was optimized through protease screening and response surface optimization. The ultrafiltration was used to separate SCPs and the whitening activity was comprehensively evaluated using radical scavenging rate and tyrosinase-inhibiting activity, among others. An optimal component was characterized and identified using various modern spectral analysis techniques. The results showed that the surface of silver carp scales after decalcification was smooth and clear. The pepsin had the highest peptide yield and tyrosinase-inhibiting activity (90.01% and 82.25%, respectively). The optimal enzymatic hydrolysis conditions were an enzyme dosage of 16.1%, a solid–liquid ratio of 1:15.6 and a time of 4.9 h. The proportions of hydrophobic and basic amino acids in the peptide composition were 32.15% and 13.12%, respectively. Compared with SCPs2, SCPs1 (6096.68–9513.70 Da) showed better ·OH scavenging ability, tyrosinase-inhibiting activity and moisture absorption. SCPs1 was a macromolecular fragment of type I collagen with a triple helix structure, containing three peptide sequences with the potential for tyrosinase activity inhibition (AGPPGADGQTGQRGE, SGPAGIAGPAGPRGPAGPNGPPGKD and KRGSTGEQGSTGPLGMRGPRGAA). These results show that SCPs1 is a collagen peptide product with whitening potential.

## 1. Introduction

Silver carp (*Hypophthalmichthys molitrix*) is a popular fresh-water-farmed fish in China due to its high productivity, excellent nutritional value and inexpensive price [1,2]. However, the multitude of byproducts, such as fish scales, skin, bones and viscera, produced during the processing of silver carp are often discarded, resulting in serious environmental pollution and resource wastage [3]. As there is a high content of collagen in fish scales, utilizing it can not only reduce the damage to the environment but also increase the potential income of the aquaculture industry [4]. After enzymatic hydrolysis, collagen can form collagen peptides with different molecular sizes and certain biological activities, such as antifreeze, anti-inflammatory and hypoglycemic activity, as well as other functionally active peptides [5,6,7]. At present, the extraction of collagen peptides from natural substances has been widely studied and aquatic animals have the advantages of low resistance and allergenicity. Hence, aquatic animals have become a major source of natural collagen peptides [8].

Owing to an improvement in the standard of living, the demand for skin health and beauty is increasing [9], and related research has received much attention. These studies on using fish collagen peptides for skin health mainly focus on antioxidation, anti-aging and anti-photoaging. For example, Sierra et al. studied the antioxidant activity of collagen peptides from Oreochromis sp. scales, and the results showed that collagen peptides with a molecular weight of 3–10 kDa had the highest antioxidant activity [10]. Similarly, Cesar et al. studied the functional activity of collagen peptides from different fish byproducts and determined that all components showed certain antioxidant activity [11]. Wang et al. discovered that collagen peptides from *Oreochromis niloticus* can effectively delay skin aging [12], and Xu et al. reported that low molecular weight skipjack tuna skin collagen peptides had an improvement effect on skin photoaging and were expected to be developed as new functional foods [13]. However, there has been relatively little research on the effects of fish collagen peptides on skin whitening.

Therefore, the intention of this study was to prepare scale collagen peptides (SCPs) with whitening activity from silver carp scales, and to optimize the enzymatic hydrolysis process using single-factor experiments and the Box–Behnken design (BBD). After characterization via amino acid analysis, the fish scale hydrolysate was separated and purified using ultrafiltration (ultrafiltration membrane filtrate cut-off = 10 kDa). The separated components were evaluated for whitening activity by comprehensive evaluation of tyrosinase-inhibiting activity, free radical scavenging ability, moisture absorption, etc. Fourier-transform infrared spectroscopy (FTIR), X-ray diffraction spectroscopy (XRD), matrix-assisted laser desorption ionization time-of-flight mass spectrometry (MALDI-TOF-MS) and ultra-performance liquid chromatography-tandem mass spectrometry (UPLC-MS/MS) were used to characterize and identify the optimal components possessing whitening activity.

## 2. Materials and Methods

### 2.1. Materials and Reagents

Silver carp scales were supported by Qianjiang Changui Aquatic Food Co., Ltd. (Qianjiang, China). Pepsin (1200 U/g), H_2_O_2_ and FeSO_4_·7H_2_O were purchased from Sinopharm Chemical Reagent Co., Ltd. (Shanghai, China). Alkaline protease (200 U/μL) was provided by Wuxi Youpuke Biotechnology Co., Ltd. (Wuxi, China). Neutral protease (50 U/mg), reduced glutathione, trichloroacetic acid (TCA), L-tyrosine, tyrosinase, DPPH and other reagents were purchased from Shanghai Macklin Biochemical Co., Ltd. (Shanghai, China). Commercial tilapia SCPs (CAPs) (MW = 600–1500 Da) were purchased from Hainan Huayan Collagen Technology Co., Ltd. (Hainan, China). All reagents were analytically pure.

### 2.2. Fish Scale Pre-Treatment and Principal Component Determination

Impurities in fish scales were picked out, and then the scales were washed and dried in an oven (DHG-9203A, Shanghai Yi Heng Co., Ltd., Shanghai, China) at 50 °C. Drying [14], muffle furnace ashing (AOAC938.08, 2007), Soxhlet extraction (AOAC960.39, 2007) and the Kjeldahl method [15] were used to determine the moisture, ash, fat and protein contents, respectively. After weighing, the dried fish scales, a 4% (*w*/*v*) hydrochloric acid solution was added at a solid-liquid ratio of 1:50. The mixture was stirred for 6 h with a stirrer for decalcification. The decalcified fish scales were washed repeatedly with running water until the wash solution was neutral, then dried in a 50 °C oven for later use. The structural changes in the fish scales after decalcification were observed and analyzed using scanning electron microscope (SEM) (SU8100, Hitachi Ltd., Tokyo, Japan).

### 2.3. Optimization of Enzymatic Hydrolysis Process

#### 2.3.1. Screening of Proteases

Neutral protease, alkaline protease, pepsin and a mixture of alkaline protease and pepsin (1:1) were tested for peptide yield and tyrosinase-inhibiting activity to select the optimal hydrolysis enzyme. Identical enzyme dosage (10%) of protease were added, and 10 g of fish scales were enzymolyzed at a solid-liquid ratio of 1:20 for 4 h under the optimal conditions for each protease. Then, the mixtures were placed in a boiling water bath at 100 °C for 20 min to inactivate the enzymes and centrifuged (TGL-24MC, Changsha Pingfan Instrument Co., Ltd., Changsha, China) at 7155× *g* for 25 min at 18 °C. The supernatant was collected, the pH was adjusted to neutral, and suction filtration was used to obtain the filtrate. Each filtrate was transferred into individual 200 mL volumetric flasks and diluted to obtain a collagen peptide enzymatic hydrolysate.

#### 2.3.2. Single-Factor Experiments and Response Surface Optimization of Enzymatic Hydrolysis

The optimal protease was selected and the effects of the enzyme dosage, solid-liquid ratio and enzymatic hydrolysis time on the peptide yield were analyzed. The single-factor experiments were implemented sequentially at 55 °C and pH 2.5. The enzyme dosage was set to 4%, 8%, 10%, 12%, 16% and 20%; the solid-liquid ratio was set to 1:12, 1:14, 1:16, 1:18 and 1:20; and the enzymatic hydrolysis time was set to 3, 4, 5, 6 and 7 h. In the first single-factor experiment, the solid-liquid ratio and enzymatic hydrolysis time were tentatively set at 1:16 and 5 h, respectively. Based on the results, the relevant parameters were fixed sequentially. In accordance with the single-factor experiment, with the BBD of Design-Expert 8.0.6 software (Stat-Ease, Inc., Minneapolis, MN, USA) and the inhibition rate of tyrosinase activity as the index, three factors (enzyme dosage, solid-liquid ratio and enzymatic hydrolysis time) and three levels were tested to optimize the extraction of SCPs and determine the final enzymatic hydrolysis conditions. The BBD is shown in Table 1.

### 2.4. Separation and Purification of SCPs

LNG-NF-103 nanofiltration membrane equipment and S-UF10 polyethersulfone resin ultrafiltration membrane (Langjimofenli Equipment Engineering Co., Ltd., Shanghai, China) was used for ultrafiltration with a molecular weight cut-off of 10 kDa. Two components containing SCPs were obtained based on their molecular weight; the fraction with molecular weight less than 10 kDa was named SCPs1 and that with molecular weight greater than 10 kDa was named SCPs2. Each component was concentrated via rotary evaporation, desalted using dialysis and freeze-dried. The whitening activity of the components was comprehensively evaluated based on their DPPH free radical inhibition rate, tyrosinase-inhibiting activity, moisture absorption and moisture retention. The selected fraction was further characterized to determine its structure and peptide sequence.

### 2.5. Determination of Peptide Yield

The peptide yield was determined using a published protocol [16] with a slight modification. Glutathione solution (0, 0.3, 0.6, 0.9, 1.2 and 1.5 mg/mL) was used as the standard, and 3 mL of the standard solution was mixed with 2 mL dithiothreitol (DTT). After the mixture was left to stand for 10 min, the optical density (OD) was measured at 540 nm using a UV spectrophotometer (UH5300, Hitachi Ltd., Tokyo, Japan). The standard curve y = 0.0595x − 0.001 (*R*^2^ = 0.9951) was obtained. Then, 5 mL fish scale enzymatic hydrolysate was mixed with 5 mL of 10% TCA solution, and the mixture was homogenized using a WH-2 vortex mixer (Shanghai Huxi Analysis Instrument Factory Co., Ltd., Shanghai, China). After allowing the mixture to stand for 10 min, it was centrifuged at 1777× *g* for 10 min. The supernatant was diluted 100-fold with 5% TCA solution. After mixing, the OD value was measured at 540 nm using a UV spectrophotometer, with the zero tube of the standard used as the blank control. The peptide yield was calculated using the following formula:

(1) $$\mathrm{Peptide}\;\mathrm{yield}\;(\%)=\frac{c\;\times \;n\;\times \;v}{m}\;\times \;100\%,$$

where c is the peptide mass concentration in the enzymatic hydrolysate (g/mL), v is the solution volume (mL), n is the dilution factor and m is the total protein content in the fish scales (g).

### 2.6. Amino Acid Composition of SCPs

The SCPs enzymatic hydrolysate obtained under the optimal conditions of the response surface experiment was inactivated and centrifuged, and the supernatant was vacuum freeze-dried (FD5-2.5, GOLD SIM International Co., Ltd., Beijing, China) for later use. Based on a previously described method [17], an automatic amino acid analyzer was used to determine the amino acid composition of the freeze-dried product (L-8900A, Hitachi Ltd., Tokyo, Japan).

### 2.7. Comprehensive Evaluation of SCP Whitening Activity

#### 2.7.1. Determination of DPPH Radical Scavenging Ability

The DPPH radical scavenging activity was determined using a published protocol [18] with appropriate modifications. The DPPH radical scavenging activity of SCPs1, SCPs2 and CAP solutions was measured at concentrations of 1, 5, 10, 15 and 20 mg/mL. DPPH–ethanol solution (0.10 mmol/L, 100 μL) and the sample solutions at different concentrations (100 μL) were added in sequence, and the mixture was reacted in the dark for 20 min. The absorbance (A) value was measured at 517 nm using a UV-Vis spectrophotometer. The DPPH radical scavenging activity was calculated according to Equation (2):

(2) $$\mathrm{DPPH}\;\mathrm{radical}\;\mathrm{scavenging}\;\mathrm{ability}\;(\%)=\left(1\;-\frac{A_{x\;}-{\;A}_{0}}{A_{1}}\right)\;\times \;100\%,$$

where A_x_, A_0_ and A_1_ are the absorbance values of the sample, the control and the distilled water blank control, respectively.

#### 2.7.2. Determination of Hydroxyl Radical Scavenging Rate

The hydroxyl radical scavenging rate was determined using a published protocol [19] with a slight modification. Each sample solution (1 mL) at concentrations of 1, 5, 10, 15 and 20 mg/mL was added to a colorimetric tube containing 8.8 mmol/L H_2_O_2_ and 9 mmol/L FeSO_4_; then, 9 mmol/L salicylic acid-ethanol solution was added, and the tube was shaken well and placed in a water bath at 37 °C for 30 min. The absorbance (A) value of each sample was measured at 510 nm. The hydroxyl radical scavenging rate was calculated according to Equation (3):

(3) $$\mathrm{OH}\;\mathrm{scavenging}\;\mathrm{ability}\;(\%)=\left(1\;-\frac{A_{x\;}-{\;A}_{0}}{A_{1}}\right)\;\times \;100\%,$$

where A_x_, A_0_ and A_1_ are the absorbance values of the sample, the control and the distilled water blank control, respectively.

#### 2.7.3. Determination of Tyrosinase-Inhibiting Activity

The tyrosinase-inhibiting activity was determined based on the method described by Song et al. [20] with a slight modification. The reagents were evenly mixed and transferred to a 96-well plate, which was placed in a 28 °C water bath for 15 min. The absorbance was measured at 475 nm using a microplate reader (DR-200Bs, HiwellDiatek Instruments Co., Ltd., Wuxi, China). The tyrosinase-inhibiting activity was calculated according to Equation (4):

(4) $$\mathrm{Tyrosinase}\text{-}\mathrm{inhibiting}\;\mathrm{activity}\;(\%)=\left(1\;-\frac{A_{4}-{\;A}_{3}}{A_{2}-A_{1}}\right)\;\times \;100\%,$$

where A_1_, A_2_, A_3_ and A_4_ are the absorbance of the solvent background, solvent control, sample background and sample reaction wells, respectively.

#### 2.7.4. Determination of Moisture Absorption Rate and Moisture Retention Rate

SCPs1, SCPs2 and CAPs were accurately weighed (0.1 g) into separate weighing bottles. The bottles were placed with their lids open at room temperature (RT = 28 °C) in a saturated ammonium sulfate solution dryer at a relative humidity (RH) of 81% for 2, 4, 6, 8, 10, 12, 24 and 48 h. The samples were weighed before (M_x_) and after (M_1_) placing them in the dryer, and moisture absorption was calculated using Equation (5):

(5) $$\mathrm{Moisture}\;\mathrm{absorption}\;(\%)=\frac{M_{1}-M_{x}}{M_{x}}\;\times \;100\%.$$

SCPs1, SCPs2 and CAPs were accurately weighed (0.1 g) into separate weighing bottles. Ultrapure water was added to the bottles to produce a 10% concentration aqueous solutions; then, the bottles were placed in a dryer containing dry silica gel powder for 2–48 h. The samples were weighed before (H_x_) and after (H_1_) placing them in the dryer, and moisture retention was calculated using Equation (6):

(6) $$\mathrm{Moisture}\;\mathrm{retention}\;(\%)=\frac{H_{1}}{H_{x}}\;\times \;100\%.$$

### 2.8. SCPs1 Structural Characterization and Molecular Weight Analysis

FTIR, XRD and MALDI-TOF-MS were used to characterize the structure and determine the molecular weight of the optimal component, SCPs1.

The method used was a modified version of that followed by Wang et al. [21]. SCPs1 sample powder (1 mg) was mixed with KBr to prepare one pellets. The sample was placed in a Fourier-transform infrared spectrometer (IR Affinity, Shimadzu Inc., Tokyo, Japan,) with a wave number range of 400–4000 cm^−1^, resolution of 4 cm^−1^ and scan number of 32 to determine the secondary structure of SCPs1.

XRD analysis of the sample crystal structure was performed using an XRD instrument (D8 ADVANCE, Bruker Inc., Ettlingen, Germany) with Cu as the cathode, a voltage of 40 kV, a current of 200 mA, a scanning speed of 0.02°/min and a scanning range of 5–90° [22].

MALDI-TOF-MS (Bruker Daltonics Inc., Ettlingen, Germany) was used to determine the relative molecular weight distribution of the sample based on a modified protocol [23]. The sample was mixed with CHCA matrix in equal proportions, spotted onto a MALDI plate and air-dried; the molecular weight distribution range was measured in positive ion reflection mode, with a lens voltage of 8.02 kV and a resolution of 40,000.

### 2.9. Identification of the SCPs1 Peptide Sequence

The peptide sequence of the optimal component for whitening activity was identified using HPLC-MS/MS. An RP-C18 column (0.15 mm × 150 mm, Column Technology Inc., Fremont, CA, USA) was equilibrated with a 95% aqueous solution of 0.1% formic acid. The sample was loaded onto Zorbax 300SB-C18 peptide traps (Agilent Technologies Inc., Wilmington, DE, USA), and then separated using liquid chromatography. The liquid-phase gradient was set as follows: 0–50 min, a linear gradient of 0.1% formic acid in acetonitrile aqueous solution from 4% to 50%; 50–54 min, a linear gradient of 0.1% formic acid in acetonitrile aqueous solution from 50% to 100%; and 54–60 min, 0.1% formic acid in acetonitrile aqueous solution maintained at 100%. A Q Exactive^TM^ mass spectrometer (Thermo Fisher Scientific Inc., Bremen, Germany) was used for detection in the positive ion mode, and the analysis time was 1 h. The raw data were searched against the corresponding database using the MaxQuant 1.5.5.1 software (Max Planck Institute of Biochemistry., Martins Reid, Germany) and compared with the UniProt database for analysis. Finally, the target peptide sequence and its identification results were obtained.

### 2.10. Data Analysis

Origin 2019 (Origin Lab Co., Ltd., Northampton, MA, USA) was used for plotting data, and SPSS 26.0 software (International Business Machines Co., Ltd., Chicago, IL, USA) was used for statistical analysis. Significance tests were performed using analysis of variance, and the significant difference level was set at *p* < 0.05. The data were expressed as means ± standard deviation (Mean ± SD), and all experiments were repeated at least three times.

## 3. Results and Discussion

### 3.1. Basic Composition and Surface Microstructure of Fish Scales

**Figure 1:**
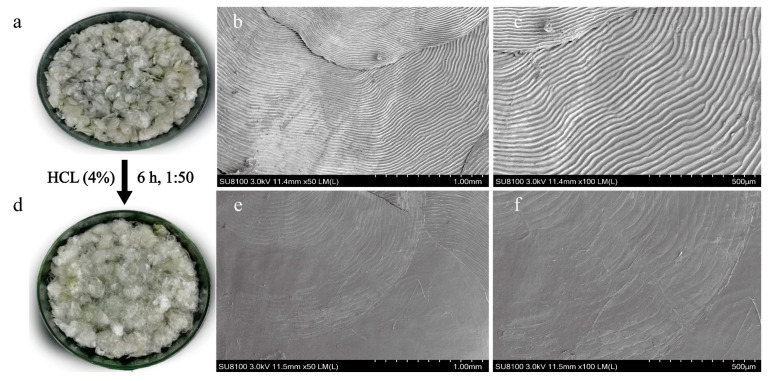
The surface microstructure changes in silver carp scales before and after decalcification. (**a**) Scale, (**b**) scale surface (×50, SEM), (**c**) scale surface (×100, SEM), (**d**) decalcified scale, (**e**) decalcified scale surface (×50, SEM), (**f**) decalcified scale surface (×100, SEM).

Basic composition analysis showed that the moisture content of carp scales was 6.26%, ash content was 19.66%, fat content was 0.93% and protein content was 74.07%. As shown in the SEM image in Figure 1, the surface of the fish scales before decalcification appeared striped with curved edges and a thick texture at the main part where hydroxyapatite was present. In contrast, the surface of the decalcified fish scales was smooth and clear, indicating that the mineral component had been removed. The ash content mainly consisted of calcium salts such as hydroxyapatite (Ca_10_(PO_4_)_6_(OH)_2_) [24], which could have a negative impact on the extraction of collagen peptides. Therefore, decalcification of the fish scales was necessary prior to extraction. The unhindered contact of collagen with the external environment is beneficial for further extraction and purification of SCPs.

### 3.2. Optimization of the Enzymatic Hydrolysis Process

**Figure 2:**
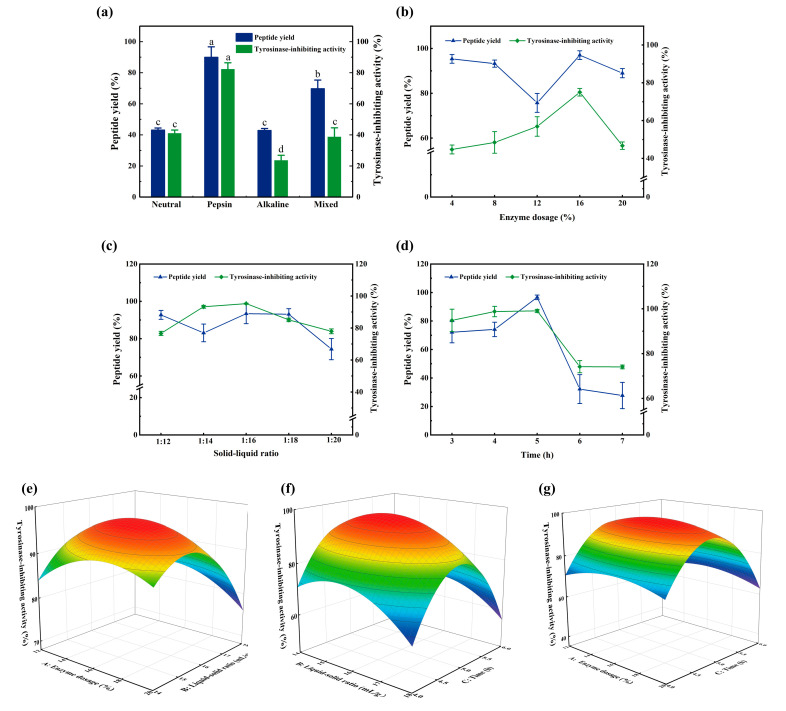
Screening of protease and optimization of enzymatic hydrolysis process. (**a**) Screening of protease; (**b**–**d**) represents the effects of enzyme dosage, solid-liquid ratio and time on peptide yield and tyrosinase-inhibiting activity; (**e**–**g**) represents the response surface 3D diagram of enzyme dosage and solid–liquid ratio, solid–liquid ratio and time, enzyme dosage and time, respectively. Different lowercase letters represent significant differences between groups (*p* < 0.05).

As shown in Figure 2a, different proteases had different effects on the hydrolysis of carp scales collagen, and the pepsin group showed the highest peptide yield and tyrosinase-inhibiting activity of 90.01% and 82.25%, respectively. Zhu et al. [25] studied the carp skin and discovered that pepsin was beneficial for the cleavage of the terminal peptide region of stable collagen molecules, thereby improving the hydrolysis efficiency. Although the pH value was neutralized after washing with HCl solution in the pre-treatment process of fish scales, residual H^+^ was still released during enzymatic hydrolysis. Pepsin hydrolysis occurred under acidic conditions (pH = 2.5), which were more conducive to maintaining hydrolysis stability. Therefore, pepsin was selected as the enzyme for fish scale enzymatic hydrolysis, and the optimal hydrolysis conditions were determined through single-factor and BBD response surface optimization experiments. BBD response surface optimization was performed using the tyrosinase-inhibiting activity as the index. Based on the results of the single-factor experiments shown in Figure 2b–d, the enzyme dosage was estimated to be 16%, the solid-liquid ratio was 1:16 and the hydrolysis time was 5 h.

Table 2 shows that the regression model of tyrosinase-inhibiting activity was significant (*p* < 0.05) and that the lack-of-fit item was not significant (*p* > 0.05). The *R*^2^ value (0.9675) indicated a high correlation between the experimental and predicted values. The model showed that *X*_2_, *X*_3_, *X*_1_^2^ and *X*_2_^2^ had a significant effect on the tyrosinase-inhibiting activity (*p* < 0.05) and that X_3_^2^ had an extremely significant effect on the same (*p* < 0.0001). The other factors did not have a significant effect (*p* > 0.05). Moreover, the order of the effects was *F*(*X*_2_) > *F*(*X*_3_) > *F*(*X*_1_), i.e., a larger *F* value signified a greater effect of the factor on the experimental index [26]. Therefore, the order of the effects of each factor on the tyrosinase-inhibiting activity of SCPs was as follows: solid–liquid ratio > hydrolysis time > enzyme dosage.

The slope of the response surface in Figure 2f is steep, whereas that in Figure 2e,g is relatively lower. The slope of the response surface reflects the sensitivity of the response value to the treatment conditions [27]. Figure 2 indicates that changes in the enzymatic digestion time and solid-liquid ratio have a greater impact on the response value (tyrosinase-inhibiting activity), whereas the effect of enzyme dosage is relatively small, consistent with the results of variance analysis in Table 2. Response surface analysis indicated that the optimal enzymatic digestion conditions are an enzyme content of 16.14%, a solid-liquid ratio of 1:15.6 and an enzymatic digestion time of 4.9 h, resulting in a tyrosinase-inhibiting activity of 97.64%. The difference between the verification experiment and the theoretical prediction model was small, and the tyrosinase-inhibiting activity was 96.2%.

### 3.3. Amino Acid Composition of SCPs

Table 3 shows that the total content of glycine, proline, glutamic acid and alanine, which are the main amino acids of SCPs, is as high as 50.39%, whereas that of aromatic amino acids, such as tyrosine and phenylalanine, is only 3.49%. This verified the results of the study by Chen et al. on the main amino acids in the enzymatic hydrolysate of *Andrias davidianus* skin [28]. The contents of hydroxyproline, basic amino acids and hydrophobic amino acids in SCPs were 9.11%, 13.12% and 32.15%, respectively. Hydroxyproline is a characteristic amino acid of collagen [29]. Basic amino acids, such as arginine and lysine, have basic groups such as protonatable guanidinium and amino groups in their side chains and hence exhibit strong free radical scavenging ability [30]. The presence of hydrophobic amino acids can increase the tyrosinase-inhibiting activity [31].

### 3.4. Whitening Activity of SCPs

**Figure 3:**
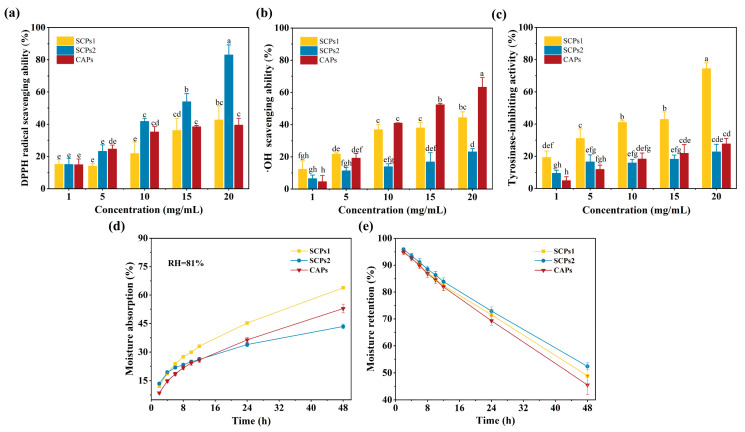
Comparison of whitening activities of each component (SCPs1 and SCPs2) after ultrafiltration. (**a**–**e**) Represent the effects of each component on DPPH radical scavenging ability, ·OH scavenging ability, tyrosinase-inhibiting activity, moisture absorption and moisture retention, respectively. Different lowercase letters indicate significant differences between groups (*p* < 0.05).

Figure 3a–d show that all indicator values increase with sample concentration. Among them, the DPPH radical scavenging ability of SCPs2 shows the most significant upward trend and is significantly different from those of SCPs1 and CAPs (*p* < 0.05). However, the ·OH scavenging rate, tyrosinase-inhibiting activity and moisture absorption rate of SCPs1 are higher than those of SCPs2 (*p* < 0.05), which may be owing to its lower molecular weight [11]. Figure 3e shows that there is no significant difference in moisturizing effect among the components (*p* > 0.05). Tyrosinase is the main factor in the formation of melanin, causing skin darkening [32]. During melanin formation, tyrosinase requires free radicals to catalyze the synthesis of L-DOPA, thereby promoting the production of melanin [33]. The SCPs1 had the optimal tyrosinase-inhibiting activity, which may be due to the higher proportion of hydrophobic amino acids in SCPs1 than other components [34]. However, the different position of the peptide sequence of the same amino acid will also affect the tyrosinase-inhibiting activity due to the different distance from the active site of tyrosinase after folding [35]. In addition, the removal of reactive oxygen species (such as ·OH and O_2_^−^·) [36] and moisturization [37] have a certain degree of influence on skin whitening. Therefore, tyrosinase-inhibiting activity was selected as the main indicator for evaluating the whitening activity of SCPs1 in this study; the other four indicators were used as reference indicators. The results indicated that SCPs1 was the optimal component with whitening activity; hence, it was further characterized and identified.

### 3.5. Structure and Molecular Weight Range of SCPs1

**Figure 4:**
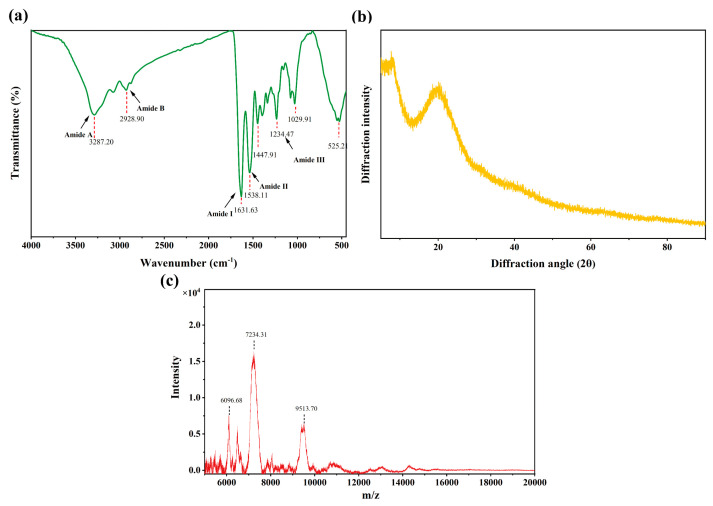
Characterization and analysis of SCPs1. (**a**) Secondary structure (FTIR); (**b**) crystal structure (XRD); (**c**) molecular weight range (MALDI-TOF-MS).

As shown in Figure 4a, SCPs1 exhibits characteristic absorption peaks in the amide A, B, I, II and III bands. These peaks conform to the peak positions of the polypeptide amide bands I and III; the peak height of the SCPs1 amide III band is 0.95 times (close to 1.0) that of the CH_2_ bending peak at 1450 cm^−1^. In the amide A and II bands, the SCPs1 absorption peak shifts to a lower frequency. Infrared spectroscopy is an effective tool for determining the secondary structure of collagen peptides [38]. Figure 4a shows that SCPs1 has the typical characteristics of fish scale type I collagen [39]. The amide A band is associated with the stretching vibration of the N-H bond. The absorption peak range of the amide A band is typically 3400–3440 cm^−1^. However, the absorption peak shifts to a lower frequency when the N-H group takes part in the formation of hydrogen bonds [40]. This indicates that SCPs1 contains hydrogen bonds and is likely to have a triple helix structure. The amide I band (1600–1700 cm^−1^) related to the stretching vibration of C=O is the main region reflecting the peptide secondary structure. The amide II band (1542–1557 cm^−1^) is generally responsible for the N-H plane bending and C-N stretching vibrations, and its peak frequency negatively correlates with the hydrogen bond content [41]. The shift of the SCPs1 amide II band peak to a lower frequency indicates an increase in the hydrogen bond content related to its N-H groups. The completeness of the collagen helix structure is generally determined by the ratio of the infrared absorption rates of the amide III band and the CH_2_ bending peak [42]. The characteristics of the SCPs1 amide III band show that pepsin has no effect on its triple helix structure.

Figure 4b shows that there are two characteristic peaks of SCPs1 at 7.76° and 20.22°. The d values of the first sharp peak and the second broad peak of the component were calculated using the Bragg equation [43] to be 11.43 and 4.21 Å, respectively. Both peaks of SCPs1 match the characteristic diffraction peaks of collagen [44]. The d value of the first sharp peak of SCPs1 results from the internal crystal of the triple helix structure of collagen. The second broad peak of SCPs1 may be related to the diffuse scattering of the collagen structure [45]. Figure 4c shows that there is no response value below 5000 m/z and no low intensity above 10,000 m/z, indicating that the molecular weight of SCPs1 mainly ranges from 6096.68 to 9513.70 Da.

These results suggest that SCPs1 is a large molecular fragment of type I collagen and retains a certain degree of the triple helix structure. Furthermore, SCPs1 may expose more hydrogen bonds. Collagen peptides inhibit tyrosinase activity mainly through hydrogen bonding and hydrophobic interactions [46]. This may account for the higher tyrosinase inhibition rate exhibited by SCPs1.

### 3.6. Active Peptide Sequence of SCPs1

The MaxQuant 1.5.5.1 software was used to search the UniProt protein database, and SCPs1 was determined to contain 39 different proteins, among which two main proteins-AOAO77B3P8 and A0A2H4ZEX8-originated from silver carp. The main peptide sequences selected from the aforementioned proteins are shown in Table 4. All these peptides belong to type I collagen, and their molecular weights are relatively high (1091.55–2359.12 Da), which is consistent with the results in Figure 4c. The biological activity of these peptide segments was predicted using the BIOPEP-UWM online database (https://biochemia.uwm.edu.pl/biopep-uwm/), URL (accessed on 22 November 2022), and the results are shown in Table 5. Three peptide segments-AGPPGADGQTGQRGE, SGPAGIAGPAGPRGPAGPNGPPGKD and KRGSTGEQGSTGPLGMRGPRGAA-with potential antioxidant activity were screened. Schurink et al. showed that arginine, proline and alanine can bind to tyrosinase, which leads to a decrease in tyrosinase activity. For example, because tyrosine is the natural substrate of tyrosinase, the similarity of proline structure to tyrosine structure thus inhibits the tyrosinase activity [47]. The presence of hydrophobic amino acids such as proline and alanine and basic amino acids such as arginine in these three peptide segments further confirm their potential to inhibit tyrosinase activity [31].

## 4. Conclusions

In this study, the sliver carp scale collagen peptides (SCPs) with whitening activity were prepared, screened and identified. The results showed that hydroxyapatite on the surface of sliver carp scales was effectively removed, and pepsin was selected as the optimal protease. Response surface optimization of the optimal enzymatic hydrolysis conditions was as follows: enzyme dosage of 16.1%, solid-liquid ratio of 1:15.6, time of 4.9 h. After ultrafiltration and comprehensive evaluation, SCPs1 was selected as the optimal whitening active component and identified as a type I collagen macromolecular fragment with a triple helix structure (Mw = 6096.68–9513.70 Da). Finally, SCPs1 screened three peptide sequences with the potential to inhibit tyrosinase activity, including AGPPGADGQTGQRGE, SGPAGIAGPAGPRGPGKD and KRGSTGEQGSTGPLGMRGPRGAA. These results provide data support for the preparation of collagen peptides with skin whitening potential.

## Figures and Tables

**Table 1:** Box–Behnken design for response surface analysis.

Level	Factor		
	*X*_1_ Enzyme Dosage/%	*X*_2_ Solid-Liquid Ratio/g/mL	*X*_3_ time/h
−1	12	1:14	4
0	16	1:16	5
1	20	1:18	6

**Table 2:** Analysis of variance of regression model.

Source	Sum of Square	Df	Means Square	*F* Value	*P*r > *F*	Significant
Model	3562.80	9	395.87	23.16	0.0002	*
*X*_1_	0.43	1	0.43	0.025	0.8784	
*X*_2_	111.31	1	111.31	6.51	0.0380	*
*X*_3_	108.51	1	18.51	6.35	0.0398	*
*X*_1×2_	26.47	1	26.47	1.55	0.2534	
*X*_1_ *X*_3_	1.96	1	1.96	0.11	0.7451	
*X*_2_ *X*_3_	3.99	1	3.99	0.23	0.6437	
*X*_1_^2^	98.85	1	98.85	5.78	0.0471	*
*X*_2_^2^	396.14	1	396.14	23.18	0.0019	*
*X*_3_^2^	2604.57	1	2604.57	152.40	<0.0001	**
Residual	119.64	7	17.09	5.77	0.0617	
Lack of Fit	97.19	3	32.40			
Pure Error	22.44	4	5.61			
Total	3682.43	16	*R*^2^ = 0.9675	*R*^2^_Adj_ = 0.9257		

Note: “*” and “**”, respectively, indicate significant difference at the level of 0.05 and 0.0001.

**Table 3:** Amino acid composition of SCPs.

Amino Acid	Residues/ mg·g^−1^	%	Amino Acid	Residues/ mg·g^−1^	%
Threonine (Thr)	23.73	3.27	Leucine (Leu) *	22.44	3.09
Serine (Ser)	28.78	3.97	Tyrosine (Tyr)	10.79	1.49
Glutamic acid (Glu)	78.78	10.86	Lysine (Lys) ^#^	24.77	3.41
Proline (Pro) *	75.19	10.37	Hlstidine (His) ^#^	11.64	1.60
Glycine (Gly)	143.35	19.76	Argnine (Arg) ^#^	58.74	8.11
Alanine (Ala) *	68.21	9.40	Isoleucine (Ile) *	14.17	1.95
Valine (Val) *	18.52	2.55	Phenylalanine (Phe) *	18.32	2.5
Methionine (Met) *	16.41	2.26	Aspartic acid (Asp)	45.43	6.3
Hydroxyproline (HyP)	66.11	9.11	Hydrophobic amino acid	233.26	32.15
			Total	725.37	100

Note: ^#^ Basic amino acid, * Hydrophobic amino acid.

**Table 4:** The main peptide sequences of SCPs1 were identified by LC-MS/MS.

No.	Sequence	Length	Mass (Da)	Score	Protein Source
1	GDKGETGEAGERGMKGHRGF	20	2074.96	268.5	Collagen type I alpha 1
2	TGPRGPVGPAGARGDKGETGEAGE	24	2222.07	258.02	Collagen type I alpha 1
3	AGPPGADGQTGQRGE	15	1396.63	254.19	Collagen type I alpha 2
4	YRGLEGNAGRDGA	13	1334.63	248.69	Collagen type I alpha 2
5	YRGLEGNAGR	10	1091.55	223.36	Collagen type I alpha 2
6	GARGDKGETGEAGERGMKGHRGF	23	2359.12	218.69	Collagen type I alpha 1
7	SGPAGIAGPAGPRGPAGPNGPPGKD	25	2151.08	218.13	Collagen type I alpha 2
8	KGETGEAGERGMKGHRGF	18	1902.91	217.68	Collagen type I alpha 1
9	KRGSTGEQGSTGPLGMRGPRGAA	23	2227.12	215.32	Collagen type I alpha 2
10	VGLTGPRGPVGPAGARGD	18	1632.87	213.73	Collagen type I alpha 1

Note: Score indicates the comprehensive score of peptide identification result. The higher the score, the more reliable the identification result.

**Table 5:** Prediction of bioactivity of main peptides in SCPs1.

No.	Sequence	Mass (Da)	Score	Bioactivity Prediction
1	AGPPGADGQTGQRGE	1396.63	254.19	antioxidative, antiamnestic, DPP-IV, ACE
2	SGPAGIAGPAGPRGPAGPNGPPGKD	2151.08	218.13	antioxidative, antithrombotic, antiamnestic, α-glucosidase inhibitor
3	KRGSTGEQGSTGPLGMRGPRGAA	2227.12	215.32	antioxidative, hypotensive, DPP-IV, ACE

## Data Availability

The data showed in this study are contained within the article.
